# *Plasmodium knowlesi* Reinfection in Human

**DOI:** 10.3201/eid1707.101295

**Published:** 2011-07

**Authors:** Yee Ling Lau, Lian Huat Tan, Lit Chein Chin, Mun Yik Fong, Mydin Abdul-Aziz Noraishah, Mahmud Rohela

**Affiliations:** Author affiliations: University of Malaya, Kuala Lumpur, Malaysia (Y.L. Lau, L.C. Chin, M.Y. Fong, M.A.-A. Noraishah, M. Rohela);; Sunway Medical Centre, Selangor, Malaysia (L.H. Tan)

**Keywords:** Plasmodium knowlesi, reinfection, malaria, virus, parasites, Malysia, letter

**To the Editor:** In 2004, a large number of patients infected with *Plasmodium knowlesi* (simian malarial species) were reported in Sarawak, Malaysia (*1*). *P. knowlesi* infection was also reported in Peninsular Malaysia (*2*).

Here we report a case of human *P. knowlesi* reinfection. Phylogenetic sequence analysis shows that the first and second infections were caused by different strains of *P. knowlesi*.

The patient was a 41-year-old businessman from Peninsular Malaysia. He was first admitted to the hospital in October 2009 with a 4-day history of fever, chills, and headache. His symptoms started ≈2 weeks after a 4-wheel-drive expedition with overnight camping in a jungle in Raub in the state of Pahang. Initial examination showed thrombocytopenia and hepatitis, and *P. knowlesi* malaria was subsequently confirmed with nested PCR by using diagnostic primers for *Plasmodium* small subunit (SSU) rRNA as described (*3*). He recovered fully after a treatment course of oral quinine plus doxycycline.

The patient was readmitted to the hospital on June 11, 2010, with a 5-day history of fever and chills and rigors, followed by epigastric pain, nausea, and vomiting. His symptoms began 15 days after another 4-wheel-drive expedition with overnight camping in a jungle in Tanjung Malim in the state of Perak. Laboratory investigations showed severe thrombocytopenia. Falciparum malaria was diagnosed initially on the basis of blood film examination with 1% parasitemia. The patient was administered oral mefloquine (750 mg) followed by 500 mg and 250 mg at 6 hours and 12 hours, respectively. His parasitemia level increased from 1.0% to 2.5% despite treatment with mefloquine. Oral quinine and doxycycline were initiated. However, renal function deteriorated further, and acute hemolysis was evident. Oral quinine was changed to intravenous quinine infusion, and oral combination of artemether and lumefantrine was added. Intermittent hemodialysis was initiated, and 1 unit each of packed erythrocyte cells and whole blood were transfused. Parasitemia eventually cleared on June 16, 2010. PCR confirmed *P. knowlesi* in the patient’s blood sample.

*P. knowlesi* has a 24-hour asexual life cycle, resulting in daily schizont rupture, which leads to high parasitemia levels. Delay in appropriate treatment, as seen in the second infection of the patient in our study, can cause severe conditions, such as thrombocytopenia, acute renal failure, and hemolysis (*4*).

To confirm the reinfection, blood samples collected from the patient at the first and second infections were reexamined. Giemsa-stained thin and thick blood films showed 2.0% and 2.5% parasitemia for the first and second infections, respectively. Some parasites showed morphologic features resembling those of *P. falciparum* ring forms and *P. malariae* trophozoite band forms.

We confirmed the *P. knowlesi* in the first and second infections by PCR, sequencing and analysis of the highly variable *csp* gene (*5*), and SSU rRNA. The nucleotide sequences of the gene were aligned by using ClustalW and analyzed by using MEGA4 software (*6*). The *csp* gene of the isolate from the first infection (denoted as Pkpah-1) was 1,217 nt, whereas the gene of the isolate from the second infection (denoted as Pkprk-1) contained 1,277 nt. This difference was due to the absence of 2 repetitive sequences (5′-gtggagcaaatgcaggacaaccgaatgcag-3′; 5′-agcaaatgcaggacaaccgaatgcagaagg-3′) in the *csp* gene of Pkpah-1. Furthermore, the alignment showed 21 variable sites between the genes. Phylogenetic analysis based on SSU rRNA sequences indicated that Pkpah-1 and Pkprk-1 formed a cluster, which was more related to *P. knowlesi* isolates from Thailand than to isolates from Sarawak, a Malaysian state in Borneo Island (Figure). Another isolate from Peninsular Malaysia, KAL-1, was also grouped with Pkpah-1 and Pkprk-1. The KAL-1 isolate was detected in a traveler from Finland returning from Peninsular Malaysia in 2007 (*7*).

**Figure:**
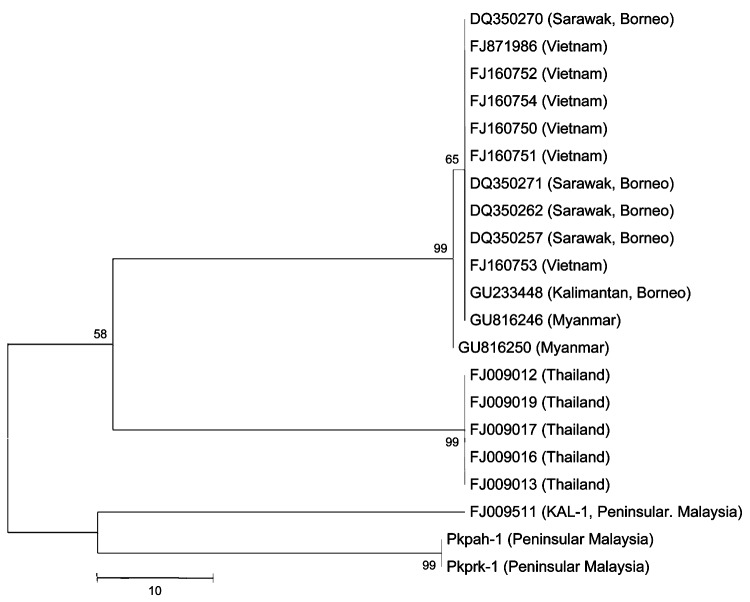
Phylogenetic trees based on nucleotide sequences of small subunit rRNA of *Plasmodium knowlesi* isolates from Peninsular Malaysia (Pkpah-1, Pkprk-1, KAL-1) and surrounding regions (denoted by GenBank accession nos.). The tree was constructed by using the maximum-parsimony method. The percentage of replicate trees in which the associated isolates cluster together in the bootstrap test (10,000 and 1,000 replicates, no differences were observed) is shown next to the branches. Phylogenetic analysis was conducted by using MEGA4 (*6*). Scale bar indicates nucleotide substitutions per site.

*P. knowlesi* infection does not relapse because the parasite has no liver hypnozoite stage (*8*). Reinfection and recrudescence are not uncommon in malaria. The finding in the patient reported here indicates reinfection rather than recrudescence as confirmed by PCR genotyping. He acquired his infection while jungle trekking in Raub in October 2009 and was reinfected in Tanjung Malim in June 2010 (the great-circle distance between the 2 locations is 38.61 km). This case shows that immunity toward *P. knowlesi* infection is strain specific as has been observed in other malaria species (*9*). *P. knowlesi* is a nonhuman primate malaria species, and humans are accidental hosts when they go into areas where macaques dwell. P. knowlesi *can cause* reinfection and can potentially be severe in areas where it is endemic. Travelers to forested areas of endemicity should be advised to take strict antimosquito measures and prophylaxis. Physicians should be aware that *P. knowlesi* infection is a vital differential diagnosis in febrile travelers with a recent travel history to forested areas in Southeast Asia.
